# Retraction: The Verbal Numerical Rating Scale and Faces Pain Scale-Revised for Children With Acute Pain: A Comparative Study for Determining the Need for Analgesia

**DOI:** 10.7759/cureus.r140

**Published:** 2024-04-26

**Authors:** Varsha Lamture, Yashwant R Lamture

**Affiliations:** 1 Pediatrics, Datta Meghe Medical College, Datta Meghe Institute of Higher Education & Research, Nagpur, IND; 2 Surgery, All India Institute of Medical Sciences, Nagpur, Nagpur, IND

This article has been retracted by the Editor-in-Chief due to concerns regarding copyright infringement of the Wong-Baker FACES® Pain Rating Scale (FACES Scale) which was modified and presented by the authors. The journal has made the decision to remove the article content in order to adhere to copyright law.

The authors agree with the decision to retract this article and remove its contents.

